# Microencapsulation of *Lactobacillus plantarum* NRRL B-1927 with Skim Milk Processed via Ultra-High-Pressure Homogenization

**DOI:** 10.3390/molecules25173863

**Published:** 2020-08-25

**Authors:** Kevin E. Mis Solval, George Cavender, Nan Jiang, Jinru Chen, Rakesh Singh

**Affiliations:** 1 Department of Food Science and Technology, The University of Georgia, Griffin, GA 30223, USA; nan.jiang6@uga.edu (N.J.); jchen@uga.edu (J.C.); 2 Department of Food Science and Technology, The University of Georgia, Athens, GA 30602, USA; cavender@uga.edu (G.C.); rsingh@uga.edu (R.S.)

**Keywords:** skim milk, ultra-high-pressure homogenization, microencapsulation, probiotics, spray drying, particle size

## Abstract

Several health benefits are associated with the consumption of probiotic foods. Lyophilized probiotic cultures are commonly used to manufacture probiotic-containing products. Spray drying (SDR) is a cost-effective process to microencapsulate probiotics. However, the high temperatures of the drying air in SDR can inactivate significant numbers of probiotic cells. Ultra-high-pressure homogenization (UHPH) processing can modify the configuration of proteins found in skim milk which may increase its protective properties as microencapsulating agent towards probiotic cells during SDR. The aim of this study was to evaluate the effect of microencapsulating probiotic *Lactobacillus plantarum* NRRL B-1927 (LP) with UHPH-treated skim milk after SDR or freeze drying (FD). Dispersions containing LP were made with either UHPH-treated (at 150 MPa or 300 MPa) or untreated skim milk and dried via concurrent SDR (CCSD), mixed-flow SDR (MXSD) or FD. Higher cell survival (%) of LP was found in powders microencapsulated with 150 MPa-treated skim milk than in those microencapsulated with non-UHPH-treated and 300 MPa-treated skim milk via FD followed by MXSD and CCSD, respectively. Increasing UHPH pressures increased the particle size of powders produced via CCSD; and reduced particle agglomeration of powders produced via MXSD and FD. This study demonstrated that UHPH processes improves the effectiveness of skim milk as a microencapsulating agent for LP, creating powders that could be used in probiotic foods.

## 1. Introduction

Multiple health benefits have been associated with the consumption of probiotic-containing foods or supplements, including the reduction and/or prevention of diarrhea, the improvement of the balance of intestinal microbiota, and the reinforcement of mucosal defenses against pathogens [1]. An increased demand for functional foods by health-conscious consumers has driven the food industry to develop alternative strategies to incorporate probiotics into foods. Since many probiotic strains are unlikely to colonize the human gastrointestinal (GI) tract via a traditional diet, the consumption of probiotic-rich and probiotic-supplemented foods has been recommended to obtain the associated benefits. This advice has driven the surge of probiotic containing products in the marketplace [2]. The majority of probiotic microorganisms used in foods are bacteria from one of two genera: *Lactobacillus* and *Bifidobacterium* [3,4]. *Lactobacillus plantarum* NRRL B-1927 (LP) is a Gram-positive, microaerophilic, lactic acid-producing, non-spore-forming, probiotic bacterium originally isolated from sauerkraut and widely used in foods as probiotic and protective culture [5,6]. However, retaining the viability of these fastidious microorganisms after the processing and storage of the probiotic foods, and their subsequent transit through the digestive tract, which exposes the cell cultures to highly acidic conditions in the stomach and to bile salts and several enzymes in the small intestine, is one of the major challenges to commercially produce probiotic-containing products with sufficiently high cell viability [7].

One effective strategy to protect probiotic cultures from harsh environmental, processing and storage conditions is microencapsulation [1]. Freeze drying (FD) has been the preferred microencapsulation method to manufacture probiotic powders for many strains, as it has the advantage of having high cell survival rates when used with the addition of microencapsulating agents or cryoprotectants such as skim milk, whey proteins, sugars, and other biopolymers [8]. However, FD is an expensive and slow process that also requires a subsequent milling step to pulverize the FD “cake” which may adversely injure the probiotic cells [9]. Meanwhile, spray drying (SDR) has been used as an alternative cost-effective method to manufacture probiotic powders, however the high drying temperatures (typically in excess of 140 °C) and high shear conditions in atomization observed during SDR inactivates significant numbers of probiotic cells due to osmotic, heat, and oxidative stresses [10]. Furthermore, cell viability of probiotics after SRD is strongly affected by not only the drying conditions, but also the individual probiotic strain and microencapsulating agent(s) involved [3]. This creates a challenge to produce spray-dried probiotic powders with high enough cell counts, as according to the U.S. Food and Drug Administration [11], probiotic foods should have a minimum of 10^6^ colony forming units (CFU) of viable probiotic cells per mL or g of food and that one to ten billion probiotic cells should be consumed daily if one hopes to observe health benefits [12].

The use of compatible microencapsulating agents has been a strategy to potentially improve the cell survival of probiotics after SDR. Due to its nutritional and functional properties (e.g., gelation and emulsification), skim milk has been used to microencapsulate probiotics. High probiotic cell viability has been reported when reconstituted skim milk was used as a microencapsulating agent, and it is thought that this is because of the lactose and various milk proteins (caseins, α-lactoglobulin, β-lactoglobulin, bovine serum albumin, lactoferrin among others) having the ability to protect bacterial cellular structure and functions during dehydration [13]. It is also been reported that lactose can interact with the polar head groups of phospholipids and proteins of bacterial cell membrane while milk proteins are able to reduce cell membrane leakage and help maintain the integrity of the probiotic cell, thereby minimizing damage and cell inactivation during SDR [14,15]. For example, Ananta et al. [16] reported a cell survival rate as high as 60% for *L. rhamnosus* GG when it was microencapsulated with reconstituted skim milk via concurrent-spray drying (CCSD) at an outlet temperature of 80 °C, while the survival of *L. casei* Shirota microencapsulated with 30% reconstituted skim milk was 94% after CCSD at outlet temperature ranging between 64–68 °C [17]. This effect is not always seen across strains, as a cell survival of only 10% was reported when *L. plantarum* CIDCA 83114 (isolated from kefir) was microencapsulated with reconstituted skim milk via CCSD at an outlet temperature of 70 °C [18]. While CCSD has been the preferred spray drying configuration to produce probiotic powders, our group has recently reported that mixed-flow spray drying (MXSD) may be a more effective method to produce spray-dried probiotic powders [19].

Ultra-high-pressure homogenization (UHPH) is a novel technology that forces liquids through a valve at pressures higher than 100 MPa. This exposes liquids to high turbulence, hydrodynamic cavitation, impingement against static surfaces, and extreme shear that can inactivate microbes, modify proteins, encourage the formation of intermolecular complexes, reduce droplet sizes and increase emulsion stability, while maintaining the nutritional and sensory qualities of the processed products [20,21,22,23,24,25]. UHPH dissociates casein micelles and partial denatures whey proteins in various ways. Sandra and Dalgleish [26] reported that treating skim milk with UHPH at 186 MPa modifies the casein micelle surface and induces calcium phosphate solubilization due to that casein micelle modification. Moreover, treating liquid milks at high UHPH pressure levels improves the binding efficiency of various compounds, such as α-tocopherol acetate, triclosan, curcumin, vitamins, or other nutritional hydrophobic compounds [27,28].

Moreover, fermented foods produced from UHPH-treated milks had higher counts of probiotics with higher cell viability than products made with non-UHPH-treated milks [29]. We have previously reported higher cell survivals of probiotic LP microencapsulated with 150 MPa-treated whey protein isolate (WPI) compared to LP microencapsulated with non-treated WPI after SDR or FD [30]. Based on these previous studies, we hypothesize that UHPH processing can change the intrinsic properties of skim milk, including protein structure, vitamin and mineral adhesion, and bioavailability of nutrients, thereby improving the cell survival of LP during drying. As there is currently a lack of scientific literature investigating the effectiveness of using UHPH-treated skim milk to protect probiotics during microencapsulation by SDR and FD, the objective of this study was to evaluate the effect of microencapsulating LP with UHPH-treated skim milk via CCSD, MXSD and/or FD.

## 2. Results and Discussion

### 2.1. Survibability of LP Cells after Drying

Cell counts of LP in dispersions prepared with non-UHPH-treated and UHPH-treated skim milk ranged between 8.81 to 9.28 Log CFU/g of solids (Table 1). After drying, significantly (*p* < 0.05) greater Log reductions of LP cells were observed in powders microencapsulated with non-UHPH-treated skim milk than in powders microencapsulated with UHPH-treated skim milk, which suggests that the UHPH processing modified some intrinsic properties of skim milk to a degree, which improved its protective properties towards LP cells during microencapsulation.

Interestingly, the two-way ANOVA revealed a highly significant (*p* < 0.05) interaction between the UHPH pressure level and the drying method on the cell survival (%) of LP. When treatment combinations were compared (UHPH pressure and drying method), LP powders produced with non-UHPH-treated skim milk via MXSD showed the highest Log reduction (1.28 ± 0.04, or 5.21 ± 0.55% survival), while powders microencapsulated with 150 MPa-treated skim milk via FD showed the lowest Log reductions (0.01 ± 0.02, or 97.14 ± 3.96% survival). Although the cell counts of LP were significantly reduced after drying (except for treatments prepared with UHPH-treated skim milk via FD), all of the resulting LP powders had cell counts higher than 8 Log CFU/g of solids, exceeding the FDA/industry recommendations (minimum number of viable probiotics cells of 6 Log CFU/g) for use in producing probiotic foods [31].

#### 2.1.1. Effect of UHPH Treatment on Cell Survival of LP

a. After CCSD

**Figure 1:**
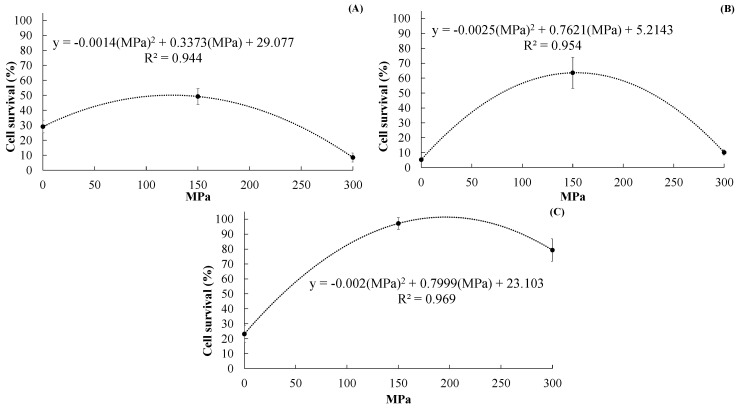
Cell survival (%) of *L. plantarum* NRRL B-1927 microencapsulated with non-UHPH-treated, 150 MPa-treated or 300 MPa-treated skim milk via CCSD (**A**), MXSD (**B**) and/or FD (**C**).

LP powders microencapsulated with 150 MPa-treated skim milk had significantly (*p* < 0.05) higher cell survival (%) than those microencapsulated with non-UHPH-treated and 300 MPa-treated skim milk (Figure 1A). Furthermore, the cell survival (%) of LP in powders prepared with 150 MPa-treated skim milk was 49.21 ± 5.31, which is 69% higher compared to powders microencapsulated with non-UHPH-treated skim milk (29.08 ± 4.23). Surprisingly, when 300 MPa-treated skim milk was used as microencapsulating agent, the cell survival of LP was reduced to 8.43 ± 2.96. To understand this effect better, an analysis of polynomial regression was developed (cell survival (%) = −0.0014 (MPa)^2^ + 0.3373 (MPa) + 29.077, R^2^ = 0.9438, *P* = 0.00007) which revealed a quadratic relationship between UHPH pressures (MPa) and cell survival (%). The regression model suggests that treating skim milk at UHPH pressures (up to ~150 MPa) increases its protective effect as microencapsulating agent for LP. However, after a certain point (~150 MPa) the intrinsic properties of skim milk are excessively modified and its protective properties as microencapsulating agent are slowly lost (denoted by the reduction of cell survival). The quadratic regression model was highly significant (*p* < 0.05) which indicates that it can be used to describe the relationship between UHPH pressures and cell survival of LP in the microencapsulated powders. These results clearly indicate that cell survival of LP after microencapsulation via CCSD can be improved by using UHPH-treated skim milk.

b. After MXSD

The cell survival (%) of LP in powders produced via MXSD are shown in Figure 1B. As in the case of CCSD, significantly (*p* < 0.05) higher cell survival (%) was obtained in LP powders microencapsulated with 150 MPa-treated skim milk than in powders produced with 300 MPa-treated and non-UHPH-treated skim milk. The cell survival (%) in LP powders prepared with 150 MPa-treated skim milk was 63.60 ± 10.49 which is more than 12 times higher than that of LP powders prepared with non-UHPH-treated skim milk (5.21 ± 0.54). While the cell survival of LP in powders microencapsulated with 300 MPa-skim milk was 10.12 ± 1.74. An analysis of polynomial regression (cell survival (%) = −0.0025(MPa)^2^ + 0.7621(MPa) + 5.2143, R^2^ = 0.95.36, *P* = 0.00004) showed the high curvilinear or quadratic relationship between UHPH pressures (MPa) and cell survival (%) of LP in the microencapsulated powders. As in the case of CCSD, using UHPH-treated skim milk improves the survivability of LP after MXSD.

c. After FD

As in the previous cases after CCSD and MXSD, LP powders microencapsulated with 150 MPa-treated skim milk showed significantly (*p* < 0.05) higher cell survival (%) than LP powders microencapsulated with non-UHPH-treated skim milk, but the increase was higher than that seen in CCSD and MXSD (Figure 1C). Also, the cell survival (%) of LP in powders microencapsulated with 150 MPa-treated skim milk was 97.14 ± 3.96, which is more than four times higher than that of LP powders microencapsulated with non-UHPH-treated skim milk (23.10 ± 5.72). An analysis of polynomial (quadratic) regression (cell survival (%) = −0.002(MPa)^2^ + 0.7999(MPa) + 23.103), R^2^= 0.8958, *p* = 0.00001) showed the high curvilinear or quadratic relationship between UHPH pressures and cell survival of LP in the microencapsulated powders produced via FD. A similar trend was observed in CCSD and MXSD.

These results indicate that the highest gains in cell survival of LP were observed in FD followed by MXSD and CCSD when using 150-UHPH-treated skim milk. Surprisingly, using 150 MPa-treated skim milk as microencapsulating agent allowed the production of LP powders via MXSD and CCSD with higher cell counts of LP than in powders microencapsulated with non-UHPH-treated skim milk via FD. These results confirmed the initial hypothesis that UHPH processing would change the three-dimensional structure of proteins/peptides present in skim milk, affecting one or more intrinsic properties, and thereby allowing UHPH-modified proteins to better protect probiotic cells from severe drying conditions (either at high or low temperatures) compared to unmodified proteins.

Interestingly, it was observed that once the UHPH treatment goes beyond a certain level (higher than 150 MPa), the protective effect for LP from drying conditions decreases, suggesting that the intrinsic properties of the skim milk were excessively modified.

According to Desrumaux and Marcand [32], UHPH treatment reduces the sizes of droplet/micelle up to a certain level before favoring coalescence or aggregation, and previous work has indeed shown that liquid milk treated at pressures higher than 200 MPa had easily observed protein micelle aggregation [33]. High UHPH pressures can also affect the denaturation/aggregation of globular whey proteins [34]. Moreover, it has been reported that UHPH treatment of skim milk at 186 MPa subjects casein micelles to such extreme cavitation, turbulence, and shear forces that the hydrophobic and ionic interactions responsible for stabilizing the casein micelle structure are disturbed, drastically reducing their size and/or causing them to disintegrate [26]. When casein micelles, formed by β-$\alpha_{s1}$-$\alpha_{s2}$- and κ-caseins (molar ratio 4:1:4:1.3), are disintegrated at certain UHPH pressures, a release of calcium, phosphate, and amino acids as well as an increase in viscosity of skim milk may be observed due to the aggregation of smaller structures such as neo-micelles and β-lactoglobulin molecules and higher protein–protein interactions, especially at pressures higher than 300 MPa [35,36]. This may explain why the current data suggested that UHPH processing at 150 MPa increases the protective properties of skim milk when used as microencapsulating agent for LP but this protective effect is greatly reduced or eliminated when the skim milk has been processed at pressure higher than 150 MPa- excessive micelle aggregation may limit the ability of those micelles to interact with the probiotic cells.

In addition to the structural issues related to proteins, it has also been reported that UHPH can improve the bioavailability of certain nutrients and other bioactives while increasing the binding efficiency of various compounds, like α-tocopherol acetate, triclosan, curcumin, vitamins, or other nutritional hydrophobic compounds, to casein proteins [27,28,37]. Skim milk contains lactose, vitamins (A and D) and minerals (especially Ca, K and Na) that are essential to support the growth of *L. plantarum*, and if these nutrients are more available during rehydration and growth, skim milk may provide better protection to probiotic bacterial cells. In fact, it has been reported that lactose increases the protective ability of skim milk during spray drying of probiotics by interacting with the cell membrane and helping it retain its structural integrity [38], perhaps through its ability to decrease the gel to liquid crystalline state transition temperature of cell membrane’s lipid bilayer [39].

It is also well known that partially digested proteins, protein hydrolysates and free amino acids are better at supporting the growth and survival of LP compared to whole proteins [6]. If UHPH creates similar products, the changes in nutrient availability may also aid the survival of the cells during drying and/or aid in the recovery of sublethally injured cells. Similar survival results have also been reported previously by our group using powders microencapsulated with UHPH-treated protein isolate encapsulants compared to those microencapsulated with non-UHPH-treated encapsulants [30].

#### 2.1.2. Effect of Drying Method on Cell Survival

LP powders microencapsulated with non-UHPH-treated skim milk showed significantly (*p* < 0.05) higher cell survival (%) after CCSD (29.08 ± 4.236), than after FD (23.10 ± 5.72), and MXSD (5.21 ± 0.55) (Figure 1). Meanwhile the cell survival (%) of LP after FD was 97.14 ± 3.96, which was significantly higher than after MXSD and CCSD, which were 63.60 ± 10.49 and 49.21 ± 5.31, respectively. When LP powders were microencapsulated with 150 MPa-treated skim milk. When LP cells were microencapsulated with 300 MPa-treated skim milk, the cell survival rates (%) of LP after FD and MXSD were 79.29 ± 7.52 and 10.12 ± 1.74, respectively. These results indicated that UHPH-treated skim milk was better at protecting LP cells during drying than non-UHPH-treated skim milk, and in some cases the survivability was either equal to or in excess of the rates seen in LP powders microencapsulated with non-UHPH-treated skim milk via FD.

FD has been the preferred method to microencapsulate probiotics because it is a gentle process that avoids thermal inactivation of probiotics’ cells, while the high temperatures used to spray dry probiotic dispersions can thermally inactivate significant numbers, and reduce the viability, of probiotic cells due to osmotic, heat, and oxidative stresses caused by the atomization of probiotic dispersions and high temperatures of the drying air [10,40]. Drying via MXSD and CCSD result in significant culture reduction due to the high drying temperatures damaging the cell wall, cytoplasmic membrane, DNA and RNA of probiotic cells, all of which can result in cell inactivation [41]. As FD is a much more expensive and slower process compared to MXSD or CCSD, current efforts are focused on improving the survivability of probiotic cells after SDR. Due to different contact configurations between the hot drying air and atomized droplets, the effects of MXSD and CCSD on the cell survival rate are different. It has been recently reported that MXSD is a faster and less severe method than CCSD to microencapsulate probiotics [19]. In this study, MXSD yielded higher cell survival than CCSD (using 150 MPa-treated skim milk) and the cell survivability of LP was tremendously improved in powders microencapsulated with UHPH-treated skim milk produced via MXSD and CCSD.

### 2.2. Moisture Content and Water Activity

The results show that LP powders microencapsulated with 150 MPa-treated skim milk have significantly (*p* < 0.05) less moisture than those microencapsulated with both 300 MPa-treated skim milk and non-UHPH-treated skim milk (Table 2).

Interestingly, the reductions in moisture content among the LP powders microencapsulated with 150 MPa-treated skim milk varied, with reductions in moisture compared with those microencapsulated with non-UHPH-treated skim milk of 33.6, 18.8, and 86.8% for CCSD, MXSD, and FD, respectively.

One potential explanation for the difference in moisture levels is that the more homogeneous LP dispersions made with the 150 MPa-treated skim milk allowed the production of smaller droplets by the spray dryer’s atomizers, which in-turn increased their surface area (relative to volume) and allowed a greater rate of evaporation inside the drying chamber of the spray dryer. Meanwhile, LP dispersions prepared with 300 MPa-treated skim milk were simply too viscous for the atomizer, which prevented the production of smaller droplets and resulted in LP powders with higher moisture content compared to those produced with 150 MPa skim milk.

The reported a_w_ values confirmed some of the moisture content observations of the LP powders. Lower a_w_ values were observed in LP powders microencapsulated with 300 MPa-treated via CCSD and MXSD. It is believed that UHPH processing increased the interaction between lactose and milk proteins which reduced the number of lactose molecules that could interact and bind with water molecules during SDR, thereby reducing the water activity of LP powders. For LP powders produced via FD, those microencapsulated with 150 MPa-skim milk had lower a_w_ values than the rest of the treatments.

As in the previous case of cell survival of LP, the interaction of UHPH level and drying method significantly (*p* < 0.05) affected both the moisture content and a_w_ values of LP powders. Water activity is an important indicator of the stability of probiotic powders. According to Abe et al., [42], higher moisture content and a_w_ values of probiotic powders are highly correlated with lower survivability and shorter shelf life. Meanwhile, Chávez and Ledeboer [43] stated that a moisture content below 5 g/100 g is necessary to guarantee the long-term stability of probiotics powders. In this study, virtually all LP powders had moisture contents below the critical value (the exception being powders produce with non-UHPH-treated skim milk via FD), which indicated that the drying conditions utilized in this project can be used to ensure the production of low-moisture LP powders.

Besides, the a_w_ values of all LP powders observed in the study were below 0.33. Probiotic powders with a_w_ values below 0.6 intrinsically prevent the growth of undesired microorganisms and result in nearly nonexistent enzymatic activity [44]. It has been reported that probiotic powders with a_w_ values between 0.15 and 0.3 can prevent caking and recrystallization of sugars such as lactose [45].

### 2.3. Particle Size Distribution

Particle size distribution of LP powders is shown in Table 3. In this study, the mean particle size (D_50_) of spray-dried powders ranged from 8.73 ± 0.08 µm (for LP powders microencapsulated with non-UHPH-treated skim milk via MXSD) to 15.76 ± 0.12 µm (for LP powders prepared with 300 MPa-treated skim milk via CCSD). Unsurprisingly, the two-way ANOVA results indicate that the particle size distribution of the LP powders was significantly (*p* < 0.05) affected by the interaction of the drying method and the level of UHPH. LP powders with significantly (*p* < 0.05) higher mean particle sizes (D_50_) were obtained via FD than with MXSD and CCSD.

According to Karam et al. [46], the grinding/milling conditions of a freeze-dried sample can affect the particle sizes of the resultant powders; while frictional heating due to excessive grinding may additionally inactivate significant numbers of probiotic cells. As the grinding conditions and equipment were the same for all FD samples in this study the differences in particle sizes among FD powders is likely the result of the intrinsic characteristics of the skim milk. Among spray-dried powders, LP powders prepared via MXSD showed significantly (*p* < 0.05) smaller mean particle sizes (D_50_) than those produced via CCSD.

It has been reported that the atomization conditions used in spray drying allows the production of small particles homogeneously distributed and the size and shapes of the resultant spray-dried particles are affected by the atomization conditions, the properties of the liquid dispersions (e.g., viscosity, pseudoplasticity, solid content, etc.), inlet and outlet air temperatures, as well as the particle residence times inside the dryer chamber [19,47].

Powders with smaller particle sizes might result from smaller droplets produced by the spray dryer’s atomizers due to the higher homogeneity of LP dispersions prepared with UHPH-treated skim milk. As previously discussed, UHPH modifies the viscosity and the structure of milk proteins, which can affect particle size distribution of the resultant LP powders. A high degree of disruption of hydrophobic and ionic interactions that results in a partial or total disintegration of the casein micelles produced by the cavitation, turbulence, and shear forces are observed at high pressures [26]. However, excessive UHPH pressures can induce the coalescence of casein micelles, increase the aggregation of smaller structures, such as neo-micelles and β-lactoglobulin molecules, and boost the protein–protein interactions, which increases the viscosity of skim milk substantially (especially at pressures higher than 300 MPa) [32,35,36].

Span value is an indicator of particle agglomeration and is an important parameter in the production of spray-dried powders. In this study, the span value of spray-dried powders ranged from 1.97 ± 0.01 (for those microencapsulated with 150 MPa-treated skim milk via CCSD) to 3.47 ± 0.06 (for those microencapsulated with non-UHPH-treated skim milk via MXSD). As expected, the span values of MXSD powders were significantly (*p* < 0.05) higher than CCSD powders, which indicated higher particle agglomeration in MXSD powders. Higher particle agglomeration have been previously reported in powders produced via MXSD than CCSD [19]. Span values higher than 2 suggests high particle agglomeration [48,49].

#### 2.3.1. Effect of UHPH Processing on Particle Size of LP Powders

**Figure 2:**
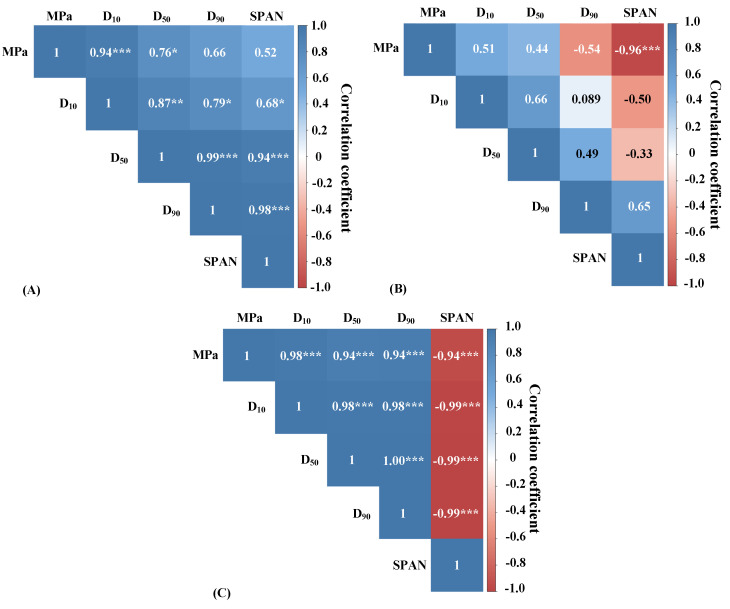
Pearson’s bivariate correlation analysis between particle size distribution values (D_10_, D_50_, D_90_, and span) and UHPH pressure level (MPa) for powders produced via CCSD (**A**), MXSD (**B**), and FD (**C**). Output numbers represent the correlation coefficients. The starts represent the *p*-value of the correlations: * *p* < 0.05, ** *p* < 0.01, *** *p* < 0.001.

Particle size is also an important quality parameter for probiotic powders, and to examine the correlation between UHPH pressures (MPa) and particle size values (D_10_, D_50_, D_90_, and span) of the resultant LP powders, a Pearson’s bivariate correlation analysis was performed (Figure 2).

A positive correlation was found between the UHPH pressures and the mean particle size (D_50_) of LP powders produced via CCSD (Figure 2A) and FD (Figure 2C). This suggests that higher UHPH pressures caused higher mean particle sizes of LP powders. This effect was more evident in LP powders produced via FD; while the mean particle sizes of LP powders produced via MXSD had no correlation with the level of UHPH processing, suggesting it was not affected.

During SDR, LP dispersions were atomized at constant conditions using a centrifugal atomizer (CCSD) or two-fluid nozzle (MXSD). Therefore, they were subjected to the same shear and mechanical stresses applied by the atomizers for a given drying type. As previously stated, UHPH processing does increase skim milk viscosity (up to certain point), which would have made more viscous LP dispersions among the UHPH-treated skim milk samples, resulting in the production of larger droplets by the atomizer used in CCSD and thereby bigger spray-dried particles. This is not unexpected, as a decreased liquid viscosity of a curcumin containing fluid was reported after treatment at 150 MPa, which in turn resulted in a reduction of both particle size and crystallinity and an increasing the water dispersity/solubility with improved bioavailability after spray drying [50].

Interestingly, in this study, lower particle agglomeration was highly correlated to the higher UHPH pressures in LP powders produced via MXSD and FD. During the atomization of liquids, dramatic increases in kinetic energy and shear force appear which increase the collision of droplets/particles at high velocities. This effect can partially damage the outer film of the droplets/particles and results in irreversible particle agglomeration [51]. Spray-dried powders with higher particle agglomeration can be solubilized/dispersed in water faster than nonagglomerated powders [52]. MXSD is a spray-drying design used to manufacture agglomerated particles which requires the use of low molecular weight sugars as “binding” agents. The obtained results suggest that more milk protein–lactose interactions occur at higher UHPH pressures thereby reducing the amount of lactose molecules available to agglomerate the dried particles.

#### 2.3.2. Correlation between Particle Size of LP Powders and Cell Survival

**Figure 3:**
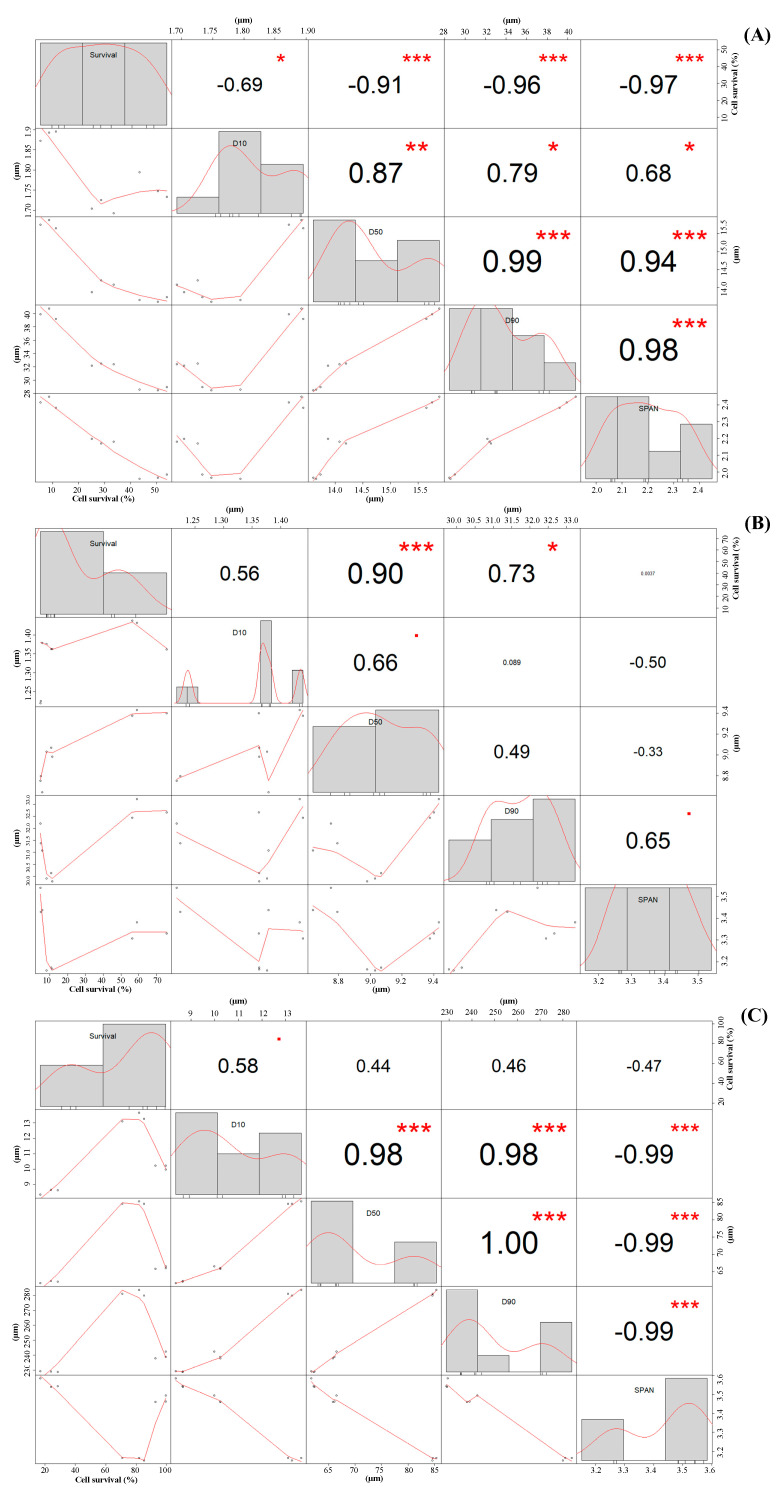
Pearson’s bivariate correlation analysis between particle size distribution values (D_10_, D_50_, D_90_, and span) and cell survival (%) for powders produced via CCSD (**A**), MXSD (**B**), and FD (**C**). Output numbers represent the correlation coefficients. The starts represent the *p*-value of the correlations: * *p* < 0.05, ** *p* < 0.01, *** *p* < 0.001.

Highly negative correlations were observed between D_50_ (−0.91 ***) and span (−0.97 ***) of LP powders produced via CCSD vs. cell survival (Figure 3A). This suggests that higher survival rates are observed in smaller and less agglomerated CCSD powders. Meanwhile, a positive correlation (0.90 ***) between D_50_ vs. cell survival in LP powders produced via MXSD (Figure 3B) was seen thereby suggesting that bigger MXSD powders had higher cell survival. Less surprisingly, no correlation was found between the particle size of powders produced via FD and cell survival (Figure 3C).

Our team has previously reported a negative correlation between the particle size of spray-dried probiotic powders microencapsulated with soy protein isolate (SPI) and/or WPI vs. the cell survival of LP [30].

Furthermore, Würth, et al. [53] have also reported a negative correlation between D_50_ of probiotic powders microencapsulated with skim milk via CCSD at 155 °C and the survival of *Lactobacillus paracasei* ssp. The authors also reported survival rates (%) of probiotics above 50% in powders with a D_50_ ≈ 5 µm, while powders with a D_50_ ≈ 10 µm had survival rates below 10%. In this study, LP powders produced via CCSD with a D_50_ ≈ 13.7 µm had cell survival (%) ~50%, while powders with D_50_ ≈ 15.8 µm showed cell survivals of ~8.5.

A final but not less important note is that this analysis of correlation between particle size of powders and cell survival does not prove causation; rather, it is used as an additional tool to understand the complexity of the results. Therefore, the high survival rates (%) observed in some LP powders may be attributed to factors other than, or in addition to their particle size.

### 2.4. Morphology of LP Powders

**Figure 4:**
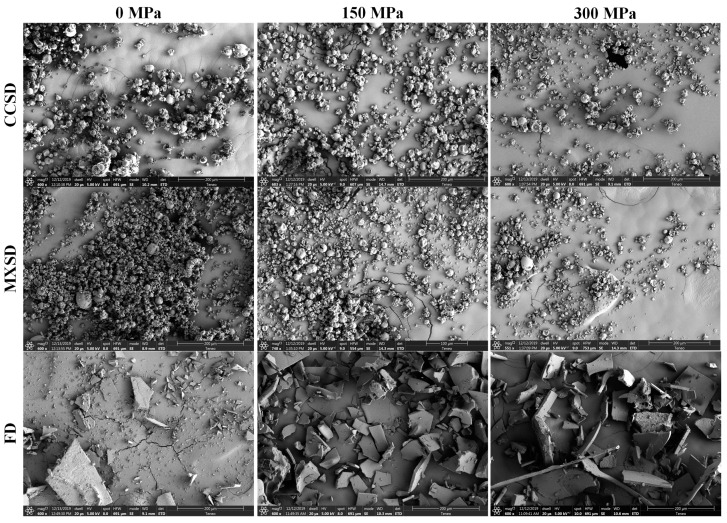
SEM micrographs of *L. plantarum* NRRL B-1927 powders microencapsulated with UHPH-treated skim milk via CCSD, MXSD and FD. Magnification is indicated on each micrograph, and varied from 550× to 750×.

The morphology of the probiotic powders was assessed by scanning electron microscopy (SEM) (Figure 4). LP powders produced via CCSD and MXSD had semispherical shapes, rough surfaces, small fissures, and shrinkage thereby suggesting a low permeability to gases and improved protection of probiotic cells [54].

The shrinkage observed in spray-dried particles produced via CCSD was higher than those produced via MXSD; which may indicate the increased severity of the former drying process. During the spray drying process, particles may shrink, inflate, agglomerate, distort or fracture depending on their composition, rheological properties, porosity degree of their skin or crust [55]. Vergara et al., [56] suggested that higher particle shrinkage may also be correlated with higher particle residence times inside the spray dryer.

In general, the particle size distribution results discussed in the previous section were confirmed with the observations in the SEM micrographs. The specific morphology of a spray-dried powder is highly related to the contact configuration between drying air and the atomized droplets, the temperature of the drying air, and the type and concentration of microencapsulating agents used [54].

Furthermore, higher degree of particle agglomeration was observed in LP powders microencapsulated with non-UHPH-treated skim milk via MXSD than CCSD. This further confirms our previous explanation of the particle size distributions of the various spray-dried LP powders. It was also noted particles of LP powders produced via FD were nonspherical, primarily flake-like with multiple spikes and sharp edges. This may had resulted from the pulverization process after the freeze-drying of the probiotic dispersions, and the particles were thus formed by multiple random fractures. The morphology of the particles of probiotic powders may use as an indicator to assess the severity of the drying process.

## 3. Materials and Methods

### 3.1. Materials

Pasteurized, grade-A fat-free skim milk was obtained from a local supermarket (The Kroger Company, Cincinnati, OH, USA), MRS broth and MRS agar from Thermo Fisher Scientific (Waltham, MA, USA), Butterfield’s phosphate buffer from Hardy Diagnostics (Santa Maria, CA, USA). A well-preserved culture sample of LP (ATCC 10241) was generously provided by the U.S. Department of Agriculture Agricultural Research Service [57] culture collection (Peoria, IL, USA). The rest of chemicals were analytical grade and were obtained from Sigma Aldrich (St. Louis, MO, USA).

### 3.2. Preparation of LP Cultures

Culture stocks of LP were prepared by following a procedure recommended by USDA-ARS [57]. LP was maintained as culture stocks in 20% glycerol (*w/v*) at −30 °C. A loop of LP culture stock was inoculated into several tubes of 9 mL of MRS broth which were incubated at 37 °C for 48 h. Afterwards, the cultures were decanted into flasks containing 3 L of MRS broth and incubated for an additional 48 h at 37 ℃. The resultant cultures were kept for up to 24 h at 4 °C before being distributed into 500 mL centrifuge bottles and pelleted using a refrigerated ultracentrifuge (Sorvall RC-6 plus, Thermo Fisher Scientific) with rotor (Fiberlite F10-6x500, Thermo Fisher Scientific) at 5000× *g* for 15 min at 4 °C. Then, the collected pellets were resuspended in 50 mL of 0.1% peptone water before combining into a single centrifuge bottle which was centrifuged a second time under the same conditions. The supernatant was then discarded, and the resultant pellet was collected and used for preparation of LP dispersions.

### 3.3. Preparation of LP Dispersions

Dispersions containing viable cells of LP were prepared by following the method previously reported by our group [30]. One liter of skim milk was subjected to UHPH processing at 150 or 300 MPa using a dual-intensifier continuous high-pressure homogenizing system (Stansted nm-gen 7900, Stansted Fluid Power, Stansted, England) that was fitted with a stainless steel metering valve (Model 60vrmm4882, Autoclave Engineers, Fluid Components, Erie, PA, USA) at the outlet and modified to feed from a 6 L vessel that was pressurized with compressed air at approximately 550 kPa. By adjusting the metering valve, the flow rate was kept at 1 ± 0.25 L/min. An unprocessed skim milk sample was used as a control. All samples were immediately cooled to below 20 °C after UHPH processing, LP pellets were then dispersed into the skim milk samples to produce LP dispersions (~9 Log CFU/g solids), which were kept at 4 °C for up to 48 h prior to drying in order to facilitate complete chilling and allow for transport between processing locations.

### 3.4. Drying of LP Dispersions

To produce probiotic powders, LP dispersions were spray dried using CCSD or MXSD conditions using a pilot-scale spray dryer (Anhydro, PSD 52, Denmark) equipped with a rotary atomizer and a two-fluid nozzle, using similar drying condition previously reported by our team [19]. In both CCSD and MXSD, the inlet air temperature was set at 140 ± 0.2 °C, while adjusting the feed flow rate between 1.5–1.75 (L/h) allowed to maintain the outlet air temperature at 80 ± 1 °C. Then, the resulting LP powders were collected and kept in a desiccator. Concurrently, LP dispersions were frozen into solid cubes (4 × 3 × 2 cm) using silicone ice trays and a freezer (Amana, Model TH21V2W, Amanda, IA, USA) with an automated temperature controller set at −4 °C for at least 12 h, then placed into a precooled pilot-scale lyophilizer (Virtis Genesis 25 ES, the Virtis Company, Gardiner, NY USA), with the temperature of the condenser and shelf initially set at −55 and −30 °C, respectively. The frozen samples were sublimated under a vacuum (<100 mTorr), for 96 h and then powdered for ~30 s. using a high-performance blender (Vitamix 7500, Olmsted Township, OH, USA). All probiotic powders were kept in a desiccator and analyzed within five days after the drying procedure.

### 3.5. Enumeration of LP Cultures

Plate counts of viable cells of LP were determined in both dispersions and powders immediately prior to and immediately after the drying process, respectively. Either 100 µL of LP dispersions or 1 g of LP powders were serially diluted in sterile Butterfield’s phosphate buffer and 100 µL of each dilution was pour plated on 100 mm diameter MRS agar plates in triplicate. Then, the inoculated plates were sealed in “zipper top” polyethylene bags (Ziploc freezer bags, S.C. Johnson & Son, Racine, WI USA) and incubated for 40 h at 37 °C under aerobic conditions before cell colonies were counted. To facilitate comparison, results were expressed as Log of colony forming units (CFU) per gram of dried solids (Log CFU/g). Cell survival rate of the probiotic cells was calculated using Equation (1)

(1) $$Cell\;survival\;\left(\%\right)=\frac{POWD}{DISP}*100$$

where, *POWD* = cell counts (CFU/g of solids) in LP powders after drying; *DISP* = cell counts (CFU/g of solids) in LP dispersions before drying.

### 3.6. Physical Properties of LP Powders

#### 3.6.1. Moisture Content and Water Activity (a_w_)

Moisture contents (g/100 g, wet basis) of the LP dispersions and LP powders were measured using a moisture analyzer (HR73 Halogen Moisture Analyzer, Mettler-Toledo GmbH, Greifensee, Switzerland). Meanwhile, the a_w_ values of LP powders were determined using a water activity meter (AquaLabSeries 3 TE, Decagon Devices, Inc., Pullman, WA, USA).

#### 3.6.2. Particle Size Distribution

Measurements for particle size distributions of the LP powders were obtained using an automated laser diffraction particle size analyzer (PSA 1190, Anton Paar GmbH, Graz, Austria). Powder samples were illuminated with three lasers from different angles with the entire light scatter pattern being collected and used to calculate the particle size distribution via a modified Michelson interferometer method. The results were reported for D_10_, D_50_, and D_90_ which are the volume diameter of the particles at 10%, 50%, and 90% cumulative volume respectively and the span value (spread of particles) was calculated by following the method referred to Mis Solval, et al. [58].

#### 3.6.3. SEM

SEM micrographs of the probiotic powders were obtained using a method previously reported by Donhowe, et al. [59]. LP powders were loaded onto a stub and sputter-coated with gold before being placed in a scanning electron microscope with an acceleration potential of 10 kV (1450 EP, Carl Zeiss MicroImaging, Inc., Thornwood, NY, USA) to observe the particle morphologies. The powders were systematically observed at a magnification between 550× and 750×.

### 3.7. Statistical Analysis

All experiments and analysis were conducted in triplicate. Means and standard deviations (SD) of test results were reported. A two-sample student’s t-test was performed to determine the significant differences between cell counts of LP before and after drying. A two-way analysis of Variance (ANOVA) (UHPH level and drying methods), and post hoc Tukey’s studentized range tests (α = 0.05) were employed to determine the statistical significance of observed differences among means using statistical software (RStudio statistical software version 1.2.5033, RStudio, Inc. Boston, MA USA). Pearson’s bivariate correlation analysis was used to (1) evaluate the correlations between UHPH pressure levels (MPa) and particle size distributions values (D_10_, D_50_, D_90_, and SPAN) of LP powders, (2) study the relationship between particle size distribution of powders and cell survival of LP.

## 4. Conclusions

The study demonstrated that the effectiveness of skim milk as microencapsulating agent for probiotic LP can be increased via UHPH processing. Using 150 MPa-treated skim milk as microencapsulating agent was more effective at increasing the cell survival of LP. Moreover, UHPH increases the protective properties of skim milk to the point that one can produce spray-dried LP powders with cell viabilities comparable to those produced via freeze-drying. In particular, UHPH-treated skim milk improves the efficacy of MXSD, a more cost-effective microencapsulation process than FD, to produce LP powders with high cell counts (8.87 ± 0.07 Log CFU/g solids) that can be used in dairy food applications. LP powders produced with UHPH-treated skim milk via CCSD had larger particle sizes and lower moisture content than those produce with non-UHPH-treated skim milk, while LP powders produced with UHPH-treated skim milk via MXSD and FD were drier and less agglomerated. Thus, the results clearly demonstrate that treating skim milk at 150 MPa improves its protective properties for the probiotic LP during drying and thereby allows the production of probiotic powders with high cell viability.

## Figures and Tables

**Table 1:** Cell counts (Log CFU/g solids) of *L. plantarum* NRRL B-1927 in liquid dispersions and powders ^†^.

UHPH	Drying Method	Log CFU/g Solids		Log Reduction
		Before Drying	After Drying	
**0 MPa**	**CCSD**	8.81 ± 0.08	8.27 ± 0.06 ***	0.54 ± 0.06 ^c^
	**MXSD**	9.23 ± 0.16	7.95 ± 0.04 ***	1.28 ± 0.04 ^a^
	**FD**	9.23 ± 0.16	8.58 ± 0.11 **	0.65 ± 0.11 ^bc^
**150 MPa**	**CCSD**	9.07 ± 0.05	8.76 ± 0.05 ***	0.31 ± 0.05 ^d^
	**MXSD**	9.07 ± 0.05	8.87 ± 0.07 **	0.20 ± 0.07 ^de^
	**FD**	9.07 ± 0.05	9.06 ± 0.02	0.01 ± 0.02 ^f^
**300 MPa**	**CCSD**	9.28 ± 0.14	8.19 ± 0.16 ***	1.09 ± 0.16 ^ab^
	**MXSD**	9.28 ± 0.14	8.28 ± 0.08 ***	1.00 ± 0.08 ^ab^
	**FD**	9.28 ± 0.14	9.18 ± 0.04	0.10 ± 0.04 ^e^

^†^ Values are means ± standard deviation (SD). *n* = 3. ^a–e^ Means with the same letter in each column are not significantly different (Tukey, *p* < 0.05). CCSD = concurrent spray drying, MXSD = mixed-flow spray drying; FD = freeze drying. Asterisks indicate significant difference between cell counts of *Lactobacillus plantarum* NRRL B-1927 (LP) before and after drying according to a two-sample student *t*-test, the starts represent the *p*-value: ** *p* < 0.01, *** *p* < 0.001.

**Table 2:** Moisture content and water activity (a_w_) values of *L. plantarum* NRRL B-1927 powders ^†^.

UHPH	Drying Method	Moisture (g/100 g, w. b.)	a_w_
**(Non-treated)** **0 MPa**	**CCSD**	3.27 ± 0.08 ^c^	0.28 ± 0.01 ^b^
	**MXSD**	4.16 ± 0.04 ^b^	0.33 ± 0.02 ^a^
	**FD**	6.69 ± 0.06 ^a^	0.13 ± 0.01 ^e^
**150 MPa**	**CCSD**	2.17 ± 0.14 ^e^	0.24 ± 0.00 ^c^
	**MXSD**	3.38 ± 0.03 ^c^	0.32 ± 0.02 ^a^
	**FD**	0.88 ± 0.04 ^g^	0.06 ± 0.01 ^f^
**300 MPa**	**CCSD**	2.46 ± 0.07 ^d^	0.23 ± 0.00 ^c^
	**MXSD**	3.23 ± 0.05 ^c^	0.25 ± 0.00 ^bc^
	**FD**	1.36 ± 0.19 ^f^	0.17 ± 0.00 ^d^

^†^ Values are means ± standard deviation (SD). *n* = 3. ^a–g^ Means with the same letter in each column are not significantly different (Tukey, *p* < 0.05). See Table 1 for description of CCSD, MXSD and FD.

**Table 3:** Particle size distribution of probiotic powders containing *L. plantarum* NRRL B-1927 ^†^.

UHPH	Drying Method	Particle Size Distribution Values			
		D_10_ (µm)	D_50_ (µm)	D_90_ (µm)	Span
**Non-treated** **(0 Mpa)**	**CCSD**	1.71 ± 0.02 ^def^	14.05 ± 0.16 ^e^	32.36 ± 0.17 ^e^	2.18 ± 0.01 ^f^
	**MXSD**	1.27 ± 0.09 ^g^	8.73 ± 0.08 ^g^	31.56 ± 0.58 ^ef^	3.47 ± 0.06 ^b^
	**FD**	8.55 ± 0.17 ^c^	62.04 ± 0.32 ^c^	229.12 ± 0.27 ^c^	3.56 ± 0.02 ^a^
**150 Mpa**	**CCSD**	1.76 ± 0.03 ^de^	13.66 ± 0.07 ^e^	28.68 ± 0.27 ^f^	1.97 ± 0.01 ^g^
	**MXSD**	1.41 ± 0.04 ^efg^	9.40 ± 0.03 ^f^	32.78 ± 0.41 ^e^	3.34 ± 0.04 ^c^
	**FD**	10.15 ± 0.15 ^b^	66.12 ± 0.36 ^b^	239.73 ± 2.37 ^b^	3.47 ± 0.02 ^ab^
**300 Mpa**	**CCSD**	1.89 ± 0.01 ^d^	15.76 ± 0.12 ^d^	39.95 ± 0.79 ^d^	2.42 ± 0.03 ^e^
	**MXSD**	1.37 ± 0.01 ^fg^	9.03 ± 0.04 ^fg^	29.94 ± 018 ^ef^	3.17 ± 0.01 ^d^
	**FD**	13.34 ± 0.28 ^a^	84.88 ± 0.42 ^a^	281.71 ± 1.99 ^a^	3.16 ± 0.01 ^d^

^†^ Values are means ± standard deviation (SD). *n* = 3. ^a–f^ Means with the same letter in each column are not significantly different (*p* < 0.05) per Tukey’s HSD post hoc testing. See Table 1 for description of CCSD, MXSD and FD.
